# Using artificial intelligence to evaluate adherence to best practices in one anastomosis gastric bypass: first steps in a real-world setting

**DOI:** 10.1007/s00464-025-11556-0

**Published:** 2025-01-27

**Authors:** Danit Dayan, Eran Nizri, Andrei Keidar

**Affiliations:** Division of General Surgery, Bariatric Unit, Tel Aviv Medical Center, Affiliated to Sackler Faculty of Medicine, Tel Aviv University, 6, Weizman St, 6423906 Tel- Aviv, Israel

**Keywords:** One anastomosis gastric bypass, Artificial intelligence, Computer vision, Safety, Best practices

## Abstract

**Background:**

Safety in one anastomosis gastric bypass (OAGB) is judged by outcomes, but it seems reasonable to utilize best practices for safety, whose performance can be evaluated and therefore improved. We aimed to test an artificial intelligence-based model in real world for the evaluation of adherence to best practices in OAGB.Please check and confirm that the authors and their respective affiliations have been correctly identified and amend if necessary.OK

**Methods:**

A retrospective single-center study of 89 consecutive OAGB videos was captured and analyzed by an artificial intelligence platform (10/2020-12/2023). The platform currently provides assessment of four elements, including bougie insertion, full division of pouch, view of Treitz ligament, and leak test performed. Two bariatric surgeons viewed all videos, categorizing these elements into Yes/No adherence. Intra-rater and inter-rater agreements were computed. The estimates found in greatest consensus were used to determine the model’s performance. Clinical data retrieval was performed.

**Results:**

Videos included primary (71.9%) and conversion (28.1%) OAGB. Patients’ age was 41.5 ± 13.6y and body mass index 42.0 ± 5.7 kg/m2. Anastomosis width was 40 mm (IQR, 30–45), and biliopancreatic limb length was 200 cm (IQR, 180–200). Operative duration was 69.1 min (IQR 55.3–97.4), mainly spent on gastric transection (26%) and anastomosis (45%). Surgeons’ intra-rater overall agreements ranged 93–100% (kappa 0.57–1). Inter-rater overall agreements increased to 99–100% (kappa 0.95–1) in the second review, set as reference point to the model. The model’s overall accuracy ranged 82–98%, sensitivity 91–94%, and positive predictive value 88–99%. Specificity ranged 17–92% and negative predictive value 20–68%.

**Conclusion:**

The model appears to have high accuracy, sensitivity, and positive predictive value for evaluating adherence to best practices for safety in OAGB. Considering the paucity of negative estimates in our study, more low-performance cases are needed to reliably define the model’s specificity and negative predictive value. Adding more best practices, tested in multi-center studies will enable cross-border standardization of the procedure.

There are many potential clinical applications of artificial intelligence (AI) in surgery [1] that will hopefully culminate in real-time decision support. The surgical video is the richest and most accurate source of data available [2]. Therefore, surgical video capturing and data analysis form the basis for novel AI-based models. The first model was described in 2006 [3]. Since then more models are being developed, using different technologies, annotations, and purposes [4–6], yet only few involve metabolic and bariatric surgery (MBS).

The Surgical Intelligence Platform, Theator Inc. (Palo Alto, California, USA) is an AI-based computer vision model that was developed, tested, and validated on thousands of surgical videos of different procedures [7, 8]. The platform performs automated data structuring into different key moments, including procedural steps, safety milestones, disease severity grading, intraoperative events, and more. Initial real-world implementation of the platform in MBS was recently described in sleeve gastrectomy [9], and we herein present the experience in one anastomosis gastric bypass (OAGB).

OAGB has gained in popularity since its first introduction by Dr. Robert Rutledge [10] and is still the third most common MBS worldwide [11]. It is recognized as an acceptable procedure by surgical associations including the International Federation for the Surgery of Obesity and Metabolic Disorders (IFSO) [12] and the American Society for Metabolic and Bariatric Surgery (ASMBS) [13] owing to accumulating evidence of sustainable optimal clinical response and low complication rate [14]. Furthermore, in a systematic review of 62,833 different bypass procedures, OAGB seemed comparably effective with better safety profile [15].

Safety in MBS is mainly judged according to outcomes (i.e., complications), but it seems reasonable to refer to objective indicators whose performance can be evaluated and therefore improved. Theator’s model was trained to assess four best practices for safety in OAGB: 1. Bougie insertion, 2. Full division of the gastric pouch from the remnant stomach, 3. View of the ligament of Treitz, and 4. Performing a leak test. Their main rationale is to create a long and narrow pouch while avoiding esophageal injury, gastro-gastric fistula, too long biliopancreatic limb, and to increase the likelihood of good sealing and patency of the gastro-jejunal anastomosis.

Our goal in this study was to test the model in real-world for the evaluation of adherence to these four best practices for safety in OAGB.

## Methods

### Setting

This is a retrospective study performed at a single tertiary bariatric center, in a large urban-public university hospital. OAGB is the most common procedure performed at our bariatric unit.

### Dataset

A total of 89 of 181 consecutive OAGB procedures (October 2020–December 2023) were fully captured and analyzed by the Surgical Intelligence Platform, Theator Inc. (Palo Alto, California, USA). All annotations and step durations were provided by the company. Ninety-two OAGB procedures were not captured by the platform due to its gradual installation on the laparoscopic towers from the initial pilot period until it was adopted for routine use or technical issues, such as hospital’s Wi-Fi disconnections.

All patients underwent a thorough workup by the multidisciplinary MBS team and were found eligible. Demographic and clinical data were retrospectively retrieved from the hospital’s electronic medical records and bariatric registry.

### AI platform

The Surgical Intelligence Platform, Theator Inc. (Palo Alto, California, USA) was used. The platform leverages proprietary AI-based computer vision to automatically capture video data during the surgery, de-identify extra-corporeal frames, and analyze the surgical video in a secure cloud infrastructure, where it is uploaded. Theator Inc. deep tech automatically identifies and generates key moments in the surgical video, providing breakdown annotations, including key steps, safety milestones, intraoperative events, and complexity scores. In all, 1730 videos were used to train (1046), test (425), and validate (259) the algorithm to recognize key moments in OAGB.

### OAGB steps

The model provides breakdown of the OAGB video into six main steps. Steps and their surgical actions include: (1) Preparation- insertion of trocars and placement of liver retractor, (2) Dissection of the lesser curvature—creating a window in the lesser omentum at or distal to the crow’s foot with an energy device, (3) Gastric transection—bougie insertion and using a linear stapling device to create a long and narrow pouch, (4) Small bowel inspection and measurement—identifying the ligament of Treitz and measuring the biliopancreatic limb, (5) Gastro-jejunal anastomosis—creating the anastomosis and performing a leak test, and (6) Final inspection—observing the operative field for hemostasis control, optional Jackson–Pratt’s drain insertion, and removal of the liver retractor and trocars.

In addition, the model recognizes Adhesiolysis as a separate step, when applicable. Irrelevant frames, such as out-of-body view recognized during the surgery itself, are de-identified by an image-blurring algorithm to maintain patient privacy and surgical team confidentiality. The out-of-body view is also time recorded by the platform.

### Best practices in OAGB

Best practices for safety in OAGB, as were defined to the model include (1) Bougie insertion, (2) Full division of pouch, (3) View of Treitz ligament, and (4) Leak test performed.

### Evaluation of the model’s performance

Two expert bariatric surgeons independently viewed and reviewed all 89 videos in two separate sessions and manually categorized all four best practices into Yes/No adherence. Full division of pouch was applicable in 88% of the videos (*n* = 78), since the remainder involved conversion of sleeve gastrectomy to OAGB. All calculations regarding full division of pouch therefore refer to percentages out of the 78 relevant videos.

Intra-rater agreements and reliability testing (i.e., kappa coefficient value) was performed—meaning that since each surgeon reviewed each case twice, we compared surgeon 1’s first assessment to his/her second assessment and surgeon 2’s first assessment to his/her second assessment. Inter-rater agreements and reliability testing was performed—meaning that we compared between surgeon 1 and surgeon 2, separately for each review assessments. Then, the AI model’s estimates were compared with the surgeons’, using their greatest consensus to formulate the model’s performance measurements, including overall agreements (i.e., accuracy) and reliability, sensitivity, specificity, and positive and negative predictive values.

### Institutional review board approval disclosure

This retrospective study was approved by the Tel-Aviv medical center Institutional Review Board (approval number 0392-22-TLV) with a waiver of informed consent according to national guidelines and was performed in accordance with the ethical standards of the institutional and /or national research committee and with the 1964 Declaration of Helsinki and its later amendments or comparable ethical standards.

### Statistical analysis

Discrete variables are presented as percentages and continuous variables are presented as mean and standard deviation or median and interquartile range (IQR). Dichotomous data were analyzed using the Chi-square test, kappa, and Fleiss’ kappa coefficient value testing. All statistical calculations were conducted using the IBM® SPSS® software platform version 29.0 and statistical significance was set at *p* < 0.05.

## Results

A total of 89 OAGB videos were automatically captured and analyzed by the AI platform. Primary OAGB comprised 71.9% (*n* = 64) and conversion OAGB comprised 28.1% (*n* = 25) of the cohort, as depicted in Table 1. Patients were predominantly females (57.3%), of mean age 41.5 ± 13.6 years and body mass index 42.0 ± 5.7 kg/m2.

**Table 1:** Patients’ baseline demographic and clinical characteristics

	*n* = 89
Age (years)	41.5 ± 13.6
Gender (females)	57.3%
Body mass index (kg/m^2^)	42.0 ± 5.7
Type 2 diabetes mellitus (*n*, %)	23 (25.8%)
Hypertension (*n*, %)	28 (31.5%)
Hyperlipidemia (*n*, %)	32 (36.0%)
Obstructive sleep apnea (*n*, %)	26 (29.2%)
Fatty liver disease (*n*, %)	68 (76.4%)
Gastroesophageal reflux disease (*n*, %)	31 (34.8%)
Primary / Conversion procedure (*n*, %)	64 (71.9%)/25 (28.1%)

Values are presented as mean ± standard deviation or percentage, as appropriate

Annotated step durations and proportions are depicted in Table 2. The total operative duration was 69.1 min (IQR 55.3–97.4), mainly spent on gastric transection and gastro-jejunal anastomosis, comprising 26% and 45% of the total time, respectively. Out of body view time was 1.2 min (IQR 0.5–2.9). Adhesiolysis was annotated in 25 videos (28.1%) and its time duration was 5.3 min (IQR 1.5–16.8).

**Table 2:** One anastomosis gastric bypass automated main steps and duration

Steps	Durations, median min (IQR)	Proportion, %
Total operation	69.1 (55.3–97.4)	
Preparation	5.4 (4.1–7.9)	8
Dissection of lesser curvature	2.8 (2.0–4.1)	8
Gastric transection	16.9 (12.8–22.9)	26
Bowel measurement	3.0 (2.3–4.3)	5
Anastomosis	29.2 (21.2–38.6)	45
Final inspection	3.8 (2.3–6.6)	6
Out of body view	1.2 (0.5–2.9)	2

Reviewing all videos, in all OAGB procedures, a long and narrow pouch was created beginning the stapling at the crow’s foot. Avoiding stapling too close to the gastroesophageal junction was verified in 72 cases (92.3% of the 78 relevant cases). The biliopancreatic limb length was 200 (IQR, 180–200) cm. The length was planned at > 200 cm and up to 250 cm in 13 cases (14.6% of the cohort), of them in 8 cases, the total bowel length was measured. 11 of the 13 cases were verified as longer than 200 cm. In contrast, in 1 case that was not planned to be > 200 cm, it was estimated as longer. Therefore, totally in 94.4% of the cohort, the limb length was created according to safe guidelines. The anastomotic width was median 40 (IQR, 30–45) mm, all ranging between 30 and 50 mm. Leak test was negative in all cases.

Focusing on safety practices that are recognized by the AI model, surgeons’ assessments of the first review were Yes in most videos for all elements: 85–88% of bougie insertion, 91–95% of full division of pouch, 83–89% of view of Treitz ligament, and 96–98% of leak test performed. Intra-rater agreements of each surgeon are depicted in Table 3. Surgeon I had test–retest overall agreements between the first and second review of 93–100% with moderate to substantial kappa values (0.57–1). Surgeon II had test–retest overall agreements between the first and second review of 95–99% with moderate to substantial kappa values (0.66–0.95). Inter-rater agreements between surgeon 1 and surgeon 2 are depicted in Table 4. Overall agreements increased from 89 to 98% in the first review session to 99–100% in the second review session, and kappa values became substantial (0.953–1.000) for all safety practices. Since surgeon 1 and surgeon 2 has the highest consensus in their second review, it was set as the reference point to the model.

**Table 3:** Surgeons’ intra-rater agreements and reliability between the first and second video evaluations

Safety component	Rater	Agreed (%)			Kappa	95% confidence interval		*p* value
		Yes	No	Overall		Lower bound	Upper bound	
Bougie insertion	Surgeon I	100	100	100	1.000	.792	1.208	< 0.001
	Surgeon II	100	99	99	.950	.742	1.158	< 0.001
Full division of pouch*	Surgeon I	99	75	95	.573	.351	.795	< 0.001
	Surgeon II	97	80	96	.707	.485	.929	< 0.001
View of Treitz ligament	Surgeon I	94	90	93	.711	.503	.919	< 0.001
	Surgeon II	95	100	95	.807	.600	.1.015	< 0.001
Leak test	Surgeon I	100	100	100	1.000	.792	.1.208	< 0.001
	Surgeon II	100	50	98	.655	.447	.863	< 0.001

^*^Full division of pouch was available in 78 videos (88%), involving conversion from sleeve gastrectomy

**Table 4:** Surgeons’ inter-rater agreements and reliability for the first and second video evaluations

Safety component	Video review	Agreed (%)			Kappa	95% confidence interval		*p* value
		Yes	No	Overall		Lower bound	Upper bound	
Bougie insertion	First	100	85	98	.904	.696	1.111	< 0.001
	Second	100	92	99	.953	.746	1.161	< 0.001
Full division of pouch*	First	95	25	91	.175	-.047	.397	0.123
	Second	100	100	100	1.000	.778	.1222	< 0.001
View of Treitz ligament	First	94	50	89	.437	.229	.644	< 0.001
	Second	100	100	100	1.000	.792	.1.208	< 0.001
Leak test	First	100	98	98	0.656	.447	.863	0.002
	Second	100	100	100	1.000	.792	.1.208	< 0.001

^*^Full division of pouch was available in 78 videos (88%), involving conversion from sleeve gastrectomy

The AI model’s estimates were then compared with the surgeons’ second review assessments to determine the model’s performance, as depicted in Table 5. The model’s overall accuracy ranged 82–98% (kappa values −0.1 to 0.74). The discrepancy between high agreements and low/absent reliability can be explained by the prevalence bias of Yes, which accounts for most estimates of surgeons’ second review and model: 86% and 81% of bougie insertion, 92% and 94% of full division of pouch, 84% and 87% of view of Treitz ligament, and 98% and 94% of leak test performed, respectively. The kappa paradox is overcome by calculations of more important measures of the model’s performance, which better differentiate between Yes and No: sensitivity which ranged 91–94% and positive predictive value of 88–99%, whereas the specificity ranged 17–92% and negative predictive value of 20–68% due to few cases of No.

**Table 5:** Comparison of the model’s accuracy, reliability, and prediction to surgeons’ assessments^§^

Safety best practices	Agreed (%)			Kappa	95% confidence interval		*p*	Sensitivity (%)	Specificity (%)	PPV (%)	NPV (%)
	Yes	No	Overall		Lower bound	Upper bound					
Bougie insertion	93	92	98	.736	.589	.883	< 0.001	93	92	99	68
Full division of pouch*	94	17	88	−.099	−.321	.123	0.384	94	17	93	20
View of Treitz ligament	91	38	82	.279	.072	.487	0.008	91	36	88	42
Leak test	94	100	94	.415	.207	.623	< 0.001	94	50	98	29

*PPV* positive predictive value, *NPV* negative predictive value

^§^Surgeons’ assessments in second video sessions were set as basis of comparison due to higher inter-rater agreements and reliability

^*^Full division of pouch was available in 78 videos (88%), involving conversion from sleeve gastrectomy

Please confirm the section headings are correctly identified.OK

Major intraoperative complications occurred in one patient (1.1%) who underwent concomitant laparoscopic splenectomy due to uncontrolled significant hemorrhage from splenic laceration. Major early (30-day) postoperative complications occurred in three patients (3.4%). Two of them developed anastomotic leak, although all intraoperative leak tests were negative. One patient underwent reoperations (for abdominal washout, drainage, and omentopexy), while the other one was treated conservatively since the leak was controlled to a Jackson–Pratt drain left during the OAGB. The third patient had anesthesia-associated complication: right pneumothorax from ventilatory barotrauma, which necessitated insertion of chest drain and was followed by surgical decortication. There were no cases of mortality.

## Discussion

Evaluating safety in OAGB involves many aspects, nevertheless, the bariatric literature is mainly narrowed down to complications [16, 17]. This is understandable, considering the simplicity of calculating complication rates. Moreover, lack of uniform technical standardization leads to wide variability in surgical data, which is abundant to begin with. Surgeons’ proficiency also plays an important role, as greater skill is associated with fewer complications [18]. Evaluating surgical performance is rendered difficult in the real world to perform by surgeons on a routine daily basis [19], not to mention time-consuming, therefore limited by low compliance and becomes impractical.

Logistic barriers are now overcome by the advent of innovative AI-based models, enhancing accessibility to well-structured automated video data that can be correlated with clinical outcomes and assist in evaluation of surgical performance by the younger generation of surgeons [20]. These insights can advance identification of best practices, which can be used for teaching and cross-border standardization of the procedure.

Defining best practices for safety is a good start. In a modified Delphi consensus on OAGB, the IFSO expert conference addressed a number of topics, some of which relate to establishing technical best practices: creating a long and narrow pouch over a bougie, stapling in the lesser curvature starts at the crow’s foot, avoiding stapling too close to the gastroesophageal junction (i.e., distancing at least 0.5–1 cm), performing a leak test, constructing anastomosis width of 3–5 cm, and measuring the biliopancreatic limb length ≤ 200 cm [21]. We found that most OAGB videos were of high safety profile in this respect, as assessed by the surgeons.

Currently though, Theator’s Surgical Intelligence Platform covers four best practices for a safe OAGB construction: bougie insertion, full division of pouch, view of Treitz ligament, and performing a leak test. The AI-based computer vision model has been trained, tested, and validated in hundreds of OAGB videos to automatically recognize and assess surgeons’ adherence to these safety indicators. Comparing model performance to assessments by bariatric surgeons, we found that the model seemed accurate, although reliability was substantial only in bougie insertion, while absent to moderate in the other three components. This is known as the kappa paradox [22]. The likely explanation lies in the prevalence bias, since most estimates were positive. This was addressed by separately calculating the model’s performance for positive and negative adherence assessments. Indeed, the model’s sensitivity ranges 91–94% and the positive predictive value 88–99%, whereas the specificity ranges 17–92% and the negative predictive value 20–68% given the few cases of No. Therefore, it appears that the model provides good detection of positive adherence with safety best practices, whereas more low safety cases are needed to determine its capability to detect negative adherence. This can be accomplished through larger cohorts that include OAGB videos performed by novice bariatric surgeons.

The added value of applying AI models in surgery is to leverage technical safety and inculcate best surgical practices among the younger generation of surgeons. The most studied safety milestone is the critical view of safety in laparoscopic cholecystectomy [23]. In a recent real-world quality initiative using the Surgical Intelligence Platform, the model drew the leadership’s attention to the low adoption rate of critical view of safety that has increased significantly through departmental intervention [24]. This sets a great example of how easy accessibility of surgical information on a routine basis enriches surgeons’ insights, motivating them to improve performance by adopting best practices for safety. In MBS, the model has been recently tested for accuracy in sleeve gastrectomy and seems promising, although more research is needed [9]. To the best of our knowledge, this is the first study that describes the model’s implementation in OAGB, which is the most common MBS in our country [25]. Currently, the model has been trained to recognize and assess four best practices in OAGB. It can be upgraded by adding more items. For example, the length of pouch—estimated by counting the number of vertical stapler firings, view of the crow’s foot upon horizontal stapling, last firing control—distancing at least 0.5–1 cm from the gastroesophageal junction that is already recognized by the model in sleeve gastrectomy, anastomosis width—by viewing the scale on the stapler upon firing, and an approximate measuring of the biliopancreatic limb length—which can perhaps be problematic to assess and teach the model. Larger multi-center cohorts are needed to further validate the model and perhaps even to mitigate technical differences, thereby promoting standardization.

The current study has several limitations. First, 92 OAGB procedures were not captured by the platform due to gradual installation on the laparoscopic towers from the initial pilot period until routine use, or technical issues, such as hospital’s Wi-Fi disconnections. However, all consecutive videos that were captured and analyzed by the platform are included in the study cohort. Second, surgeons 1 and 2 are members of the bariatric unit and performed some of the procedures that were included in the study’s cohort. This can raise a concern regarding the objectivity of surgeons’ assessments; however, it is the AI model’s performance that is under examination in the current study and not the quality of surgeons’ performance per se. Also, the elements were tested in the context of yes or no performed. We therefore believe that the chances of bias are low in this respect. Third, the prevalence bias given a majority of positive adherence to best practices; however, other measurements were performed to differentiate between the model’s performance in positive and negative adherence. More research is needed to assess reliability of negative adherence to best practices, including multi-center cohorts that involve novice bariatric surgeons. Fourth, this study focuses on adopting surgical best practices; however, there are other factors that play a role in the development of complications. Therefore, adopting best practices does not guarantee a complication-free surgery. Actually, in spite of the leak tests that were performed and found negative, two patients developed postoperative leak. More videos of complications will allow better characterization of surgeon-related factors, such as tissue handling and clinical correlations with patient-related factors. Despite that, to the best of our knowledge, this study is the first to describe real-world routine implementation of computer vision AI platform in OAGB and evaluates adherence to safety best practices and the model’s performance. Determining this is a good commence toward a cross-border process for quality standardization of OAGB. AI-based models will eventually serve as a tool for teaching and evaluating performance on a regular basis.

In conclusion, the AI-based model appears to have a satisfactory performance, with high accuracy, sensitivity and positive predictive value for evaluating adherence to four best practices for safety in OAGB. Considering the paucity of negative estimates, larger cohorts are needed to reliably define the model’s specificity and negative predictive value. Adding more best practices, tested in multi-center studies will enable cross-border standardization of the procedure.

## Data Availability

All data of this study are available within the article.
